# Laboratory-based evaluation of legionellosis epidemiology in Ontario, Canada, 1978 to 2006

**DOI:** 10.1186/1471-2334-9-68

**Published:** 2009-05-21

**Authors:** Victoria Ng, Patrick Tang, Frances Jamieson, Cyril Guyard, Donald E Low, David N Fisman

**Affiliations:** 1 Child Health Evaluative Sciences, Research Institute of the Hospital for Sick Children, Toronto, Canada; 2 National Centre for Epidemiology and Population Health, The Australian National University, Canberra, Australia; 3 Ontario Agency for Health Protection and Promotion, Toronto, Canada; 4 Department of Pathobiology and Laboratory Medicine, University of Toronto, Toronto, Canada; 5 Department of Microbiology, Mount Sinai Hospital, Toronto, Canada; 6 Department of Epidemiology, Dalla Lana School of Public Health, University of Toronto, Toronto, Canada; 7 Department of Health Policy, Management, and Evaluation, University of Toronto, Toronto, Canada

## Abstract

**Background:**

Legionellosis is a common cause of severe community acquired pneumonia and respiratory disease outbreaks. The Ontario Public Health Laboratory (OPHL) has conducted most testing for *Legionella *species in the Canadian province of Ontario since 1978, and represents a multi-decade repository of population-based data on legionellosis epidemiology. We sought to provide a laboratory-based review of the epidemiology of legionellosis in Ontario over the past 3 decades, with a focus on changing rates of disease and species associated with legionellosis during that time period.

**Methods:**

We analyzed cases that were submitted and tested positive for legionellosis from 1978 to 2006 using Poisson regression models incorporating temporal, spatial, and demographic covariates. Predictors of infection with culture-confirmed *L. pneumophila *serogroup 1 (LP1) were evaluated with logistic regression models.

**Results:**

1,401 cases of legionellosis tested positive from 1978 to 2006. As in other studies, we found a late summer to early autumn seasonality in disease occurrence with disease risk increasing with age and in males. In contrast to other studies, we found a decreasing trend in cases in the recent decade (IRR 0.93, 95% CI 0.91 to 0.95, *P*-value = 0.001); only 66% of culture-confirmed isolates were found to be LP1.

**Conclusion:**

Despite similarities with disease epidemiology in other regions, legionellosis appears to have declined in the past decade in Ontario, in contrast to trends observed in the United States and parts of Europe. Furthermore, a different range of *Legionella *species is responsible for illness, suggesting a distinctive legionellosis epidemiology in this North American region.

## Background

*Legionella *species are Gram-negative bacteria that are ubiquitous in both natural aquatic and moist soil and mud environments [1,2] and in artificial aquatic habitats [3]. Human infection with *Legionella *[4] has two distinct forms – *Legionnaires' disease*, a more severe form of infection which includes pneumonia, and *Pontiac Fever*, a milder febrile flu-like illness without pneumonia [5].

Legionellosis occurs both sporadically and in outbreaks; the latter may be community-based, hospital-based, or occur in the long-term care setting [6,7]. While major outbreaks make media headlines and prompt evaluation of point-sources of exposure [8], most legionellosis is likely sporadic. Although there are no clinical features unique to severe legionellosis [9], case fatality rates are extremely high (10–40%) and may approach 50% in nosocomial outbreaks in individuals with already compromised health status [10,11]. Sporadic cases are reported throughout the year with summer or autumn seasonality, presumably due to enhanced proliferation of the bacteria in warmer aquatic environments [11,12].

Both sporadic legionellosis, and large legionellosis outbreaks are known to occur in the Canadian province of Ontario [8,13], with over 1400 cases recorded between 1978 and 2006. However, the epidemiology of legionellosis in this region has not been reviewed previously in the biomedical literature. The centralized nature of legionellosis testing in Ontario over a thirty year period, combined with the retention of culture-based testing by the Ontario Public Health Laboratory (OPHL) in conjunction with serological and antigen-based assays for disease, make laboratory records a useful database for the evaluation of legionellosis trends in this jurisdiction since testing was first introduced. Our objectives were to provide a laboratory-based review of the epidemiology of legionellosis in Ontario; to explore the diversity of *Legionella *species that cause disease in this province; and to evaluate spatial and temporal patterns in legionellosis case occurrence over the past three decades.

## Methods

**Figure 1:**
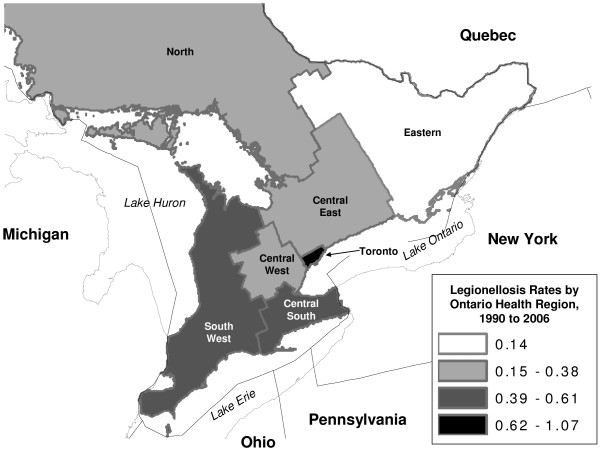
**Average Rates of Legionellosis by Ontario Health Region, 1990 to 2006**. Rates are per 100,000 persons per year, and are presented from 1990 forward due to difficulties in regional population estimation prior to that time. Note that only the southernmost extreme of the North Ontario Health Region is included on the map; this region is geographically larger than all other health regions combined, but has a far lower population density. Map scale 1 cm = 32 kilometers (1 inch = 52 miles).

Ontario is located in the east-central part of Canada and is the most populous province in Canada (population 12,160,282 in the 2006 Canadian census, or 38.5% of the Canadian population [14]). The province covers a large geographic area (917,741 km^2^), with a sparse resident population in much of northern Ontario; the most heavily populated areas of the province lie on or near the Great Lakes. Administratively, disease control activities in the province are the responsibility of 36 local public health units (PHU); however, due to a high degree of variability in PHU populations, it is convenient to aggregate these PHU into seven Ontario "health regions" (OHR), which have populations that range from approximately 0.5 to 2 million persons. These OHR include Toronto, South West, Central South, Central West, Central East, East and North (Figure 1).

Legionellosis is a notifiable disease in Ontario, with all testing for *Legionella *in the context of outbreak investigation, and most testing of clinical specimens, performed at the OPHL in Toronto. Testing records for legionellosis are available from May 1978 to the present; as such, the OPHL database contains information on most identified cases of legionellosis in Ontario over the past 30 years. The database includes information on the dates of onset of illness, case reporting, and laboratory testing; patient age and sex; and the hospital or healthcare facility from which test specimens were submitted, as well as the city or public health unit in which the submitting facility is located. Data are also available on testing type performed and test results for all patients with at least one positive test. As this is a laboratory-based database, information on city or public health unit of residence for each case, clinical characteristics and outcome, and such background data such as smoking status and or the presence of co-morbid illness, was not available.

### Case Definition and Testing Methods

Ontario adheres to the national case definition for legionellosis as defined by the Public Health Agency of Canada [15]. The culture of clinical specimens is regarded as the gold standard for diagnosis of Legionnaire's disease but immunoflourescence (direct fluorescent antibody (DFA)) and serological assays (indirect fluorescent antibody (IFA)) targeting *L. pneumophila *antigen and *L. pneumophila*-specific antibodies can also be used [5]. Immunodiagnostic methods in our laboratory involve routine testing using a large panel of reagents for both DFA and IFA; species and groups included in routine testing include *L. pneumophila *serogroups 1 to 8, *L. bozemanii, L. jordanis, L. micdadei, L. dumoffii, L. gormanii, L. oadridgensis, L. longbeachae *serogroups 1 and 2, *L. feeleii *serogroup 1, *L. anisa*, *L. wadsworthii*, and *L. maceachernii*. In outbreak situations, up to 48 *Legionella *species can be identified using IFA. Reagents are prepared following standard protocols of the U.S. Centers for Disease Control (CDC); test interpretation is according to standard procedures defined by the CDC. Potential limitations of immunodiagnostic methods include inability to detect some *Legionella *species, difficulty with validation of infection by unusual strains (due to lack of clinical material) and the potential for cross-reactivity.

A validated, in-house urine antigen immunochromatographic test (ICT) was used at the laboratory from 1984 to 2005 [16]; in 2005, poor performance of this assay in the context of a large institutional legionellosis outbreak [17] resulted in replacement of this assay with a commercially available urine ezyme-linked immunosorbent assay (Binax™).

The OPHL can receive up to three types of specimens per patient (serum, urine and respiratory tract tissues); the laboratory typically identifies and reports individuals as legionellosis cases on the basis of information derived from multiple, complementary tests (a mean of four independent tests were been performed on individuals identified as "cases" during the study period). A diagnosis of legionellosis at the OPHL was confirmed when at least one of the following criteria was met in conjunction with a compatible clinical illness: (i) isolation of a *Legionella *species or detection of the antigen from respiratory secretions, lung tissue, pleural fluid, or other normally sterile fluids; or (ii) a significant (four-fold increase or greater) rise in *Legionella *antibody titre (both IgG and IgM together) between acute and convalescent sera; or (iii) single specimen or standing *Legionella *antibody titre seroconversion from >=1:256 against *Legionella *sp.; or (iv) detection of *Legionella *soluble urine antigen [16].

### Statistical Analysis

Legionellosis rates were estimated by dividing provincial or regional disease counts by the appropriate Statistics Canada population estimates [14,18,19]. As Ontario's current public health units were defined in 1995, PHU population estimates were not available for prior years; we estimated PHU populations from 1990 to 1995 through linear extrapolation, but did not attempt regional rate calculations for years prior to 1990, due to concerns that additional extrapolation would be inaccurate.

We evaluated temporal and spatial patterns in case occurrence using Poisson regression models; seasonality was modeled by incorporating sine and cosine components into regression models (i.e., via use of the "fast Fourier transform") [20]. Models were used to estimate average rates of disease, as well as incidence rate ratios (IRR) for disease in population subgroups. Logistic regression was used to evaluate temporal changes in the likelihood that cases had been identified using different available testing methodologies.

As visual inspection of disease data suggested nonlinear changes in disease incidence over time, we evaluated both linear, quadratic, and cubic trends in incidence by incorporating year, year-squared, and year-cubed terms into regression models [21]; similar non-linear components were incorporated into logistic models for evaluation of trends in testing over time. We evaluated the improvement in model fit upon incorporation of a polynomial (i.e., squared) term into the model using the likelihood ratio test. As quadratic terms significantly improved model fit, but are complex to interpret, we present linear trends broken down into three decade-long time periods: 1978 to 1987; 1988 to 1997; and 1998 onwards; these latter estimates were generated using linear splining techniques [22]. Due to the potential for cross-reactivity of sera used in immunodiagnostic methods, we defined cases as having "definitive" speciation if the case was culture-confirmed, and "tentative" speciation if speciation was based entirely on immunodiagnostic methods. We restricted our analyses of trends and patterns of legionellosis occurrence according to species to culture-confirmed cases. Statistical analyses were performed using STATA version 9.0 (STATA Corporation, College Station, TX) and thematic maps were created using ArcGIS version 9.2 (ESRI, Redlands, CA). Due to the absence of personal identifiers in the dataset, and the fact that the study involved the use of a pre-existing dataset, the study received expedited approval from the Research Ethics Board of the Hospital for Sick Children.

## Results

### Epidemiological Profile of Legionellosis in Ontario

1401 cases of legionellosis were reported between May 1978 and December 2006; cases were identified in all health regions (Figure 1). Most cases were identified through testing performed on specimens submitted from the Toronto health region (46.5%, n = 648). The average annual number of legionellosis cases identified in the province between 1978 and 2006 was 48.3 cases (range 5 to 139), for an estimated crude average incidence of infection of 0.41 per 100,000 person years.

**Figure 2:**
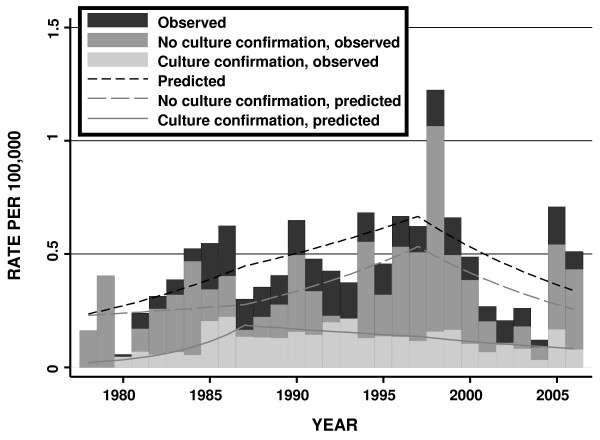
**Observed and Model-Predicted Legionellosis Rates by Culture Confirmation in Ontario from 1978 to 2006**. Bars represent observed rates of disease (black bars = overall rates; white bars = rates of disease without culture confirmation; gray bars = rates of disease with culture confirmation). Lines represent fitted regression model with spline knots occurring at approximately decade-long intervals. All rates associated with statistically significant decline since 1998.

Incidence of legionellosis peaked in late summer and early autumn, with 49% of cases (n = 688) reported during the 4-month period from July to October, and significant seasonal oscillation (*P *for log-linear combination of sine and cosine model terms < 0.001). Incidence was markedly increased in incidence among older individuals; 75% of source patients (n = 1057) were aged 50 and over (Figure 2 and Table 1). The summary incidence rate ratio (IRR) for individuals aged greater than 50 years was 9.54 (95% CI 8.41 to 10.81, *P *< 0.001). Among individuals aged 50 and over, there was a continuing, strong linear trend for increased risk with increasing age (IRR per decade increase in age 1.67, 95% CI 1.58–1.77, *P *< 0.001) (Figure 2).

Males were more likely to be infected than females, with estimated annual infection rates of 0.55 per 100,000 as compared to 0.35 per 100,000 in females (IRR 1.57, 95% CI 1.41–1.76) (Figure 2 and Table 1). Although more cases of legionellosis were reported in the oldest women than in the oldest men, this effect was due to greater numbers of women surviving to advanced age; the annualized rate of infection in the oldest men (11.2 cases per 100,000) was approximately double that seen in the oldest women (5.7 cases per 100,000) (Figure 2). The association between male gender and legionellosis risk was strengthened by adjustment for older average age in the female population (adjusted IRR 1.98, 95% CI 1.78–2.22, *P *< 0.001).

Annualized rates by OHR, during the period between 1990 and 2006 (n = 1000), ranged from 0.12 cases per year per 100,000 persons in the Eastern region to 0.87 cases per 100,000 persons in the Toronto region, with the appearance of a northeast to southwest risk gradient in the southern (most populous) part of the province (Figure 1). The highest regional rate recorded during the study period was 3.39 cases per 100,000 persons in the South West region in 1998. There was, however, a significant increase in legionellosis diagnosis in Toronto relative to non-Toronto regions (IRR 2.96, 95% CI 2.6–3.4).

### Temporal Trends in Case Occurrence

**Figure 3:**
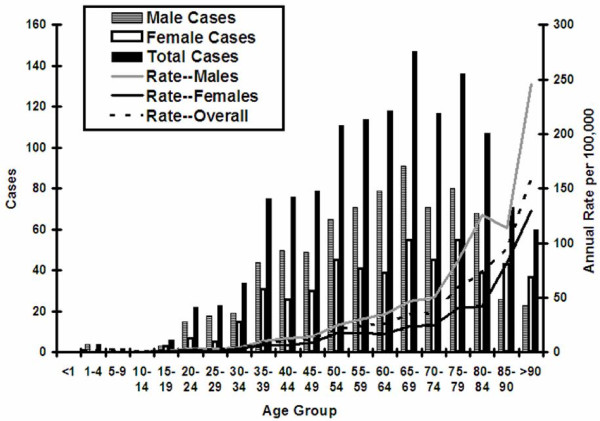
**Trends in Legionnellosis Case Counts and Rates by Age and Sex, Ontario 1978 to 2006**. A marked increase in case counts and disease rates is seen with increasing age. Diminished case counts in the oldest individuals (black bars) reflect small size of population at risk; rates increase continuously (dashed black line). Case counts in males (gray bars) are higher than those in females (white bars) in all but the oldest age groups. However, as fewer males survive to extreme old age, rates of disease per 100,000 population are higher in males (gray line) than in females (solid black line) in all age groups.

We found both graphical and statistical evidence for nonlinear changes in legionellosis incidence over time (Figure 3). Both year and year-squared terms were significantly associated with disease rates (*P *for both terms < 0.001), and the addition of a year-squared term significantly improved model fit (likelihood ratio test χ^2 ^62.0 with 1 d.f., *P *< 0.001). As such, we broke our study period into three approximately decade-long intervals, and calculated within-decade estimates of yearly trends. Disease rates increased markedly from 1978 to 1987 (IRR 1.07, 95% CI 1.04–1.11, *P *< 0.001); increased less sharply from 1988 to 1997 (IRR 1.04, 95% CI 1.02–1.06, *P *< 0.001); and declined subsequent to 1998 (IRR 0.93, 95% CI 0.91–0.95, *P *< 0.001). A decline in the most recent decade was observed even when analysis was restricted to culture-confirmed cases (IRR 0.95, 95% CI 0.90–0.99), and to cases lacking culture positivity (IRR 0.92, 95% CI 0.90–0.95) (Figure 2) and was also seen when the time series was extended to 2008 using case count data from 2007 and 2008 (IRR 0.96, 95% CI 0.94 to 0.99). From 1998 to 2006 there was no heterogeneity in observed trends according to the presence or absence of culture confirmation (P = 0.39).

### Temporal Trends in Testing

**Figure 4:**
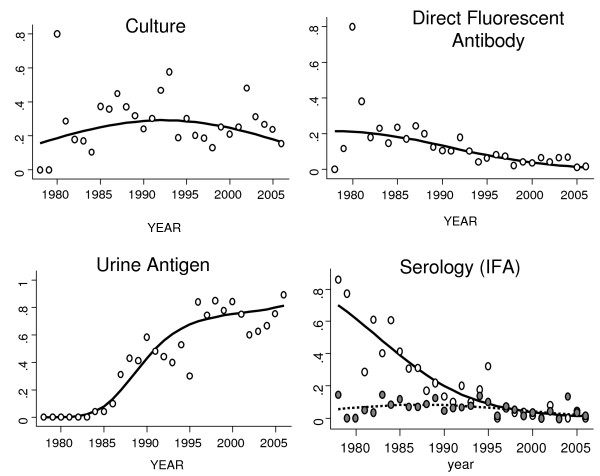
**Changing Influence of Testing Methodologies on Legionellosis Diagnosis, Ontario, 1978–2006**. Observed (dots) and expected (curves) probabilities of test positivity by methodology in 1401 cases of legionellosis diagnosed in Ontario, Canada from 1978 to 2006. In bottom right graph, solid line and white dots represent seroconversion based on paired serological testing, while broken line and gray dots represent diagnosis based on single high-titre positive serology. Expected probabilities are generated using logistic regression models, with probability of test positivity regressed against year and (in the case of culture and single high-titre immunofluorescent antibody) year-squared. Urine antigen testing was introduced at the laboratory in 1984, and consequently no specimen was positive by this method prior to that date.

There was a significant increase in the probability of culture confirmation for cases during the 1990s (OR 1.59, 95% CI 1.40–1.81%, *P *< 0.001) and a significant decrease in the probability of culture confirmation from 2000 to 2006 (OR 0.72, 95% CI 0.61–0.86, *P *< 0.001). Best fit logistic models included a quadratic term, accounting for the non-linear change in probability of culture confirmation over time. By contrast, there was a progressive increase in the likelihood of cases having a positive urine antigen test result over the entire study period (OR per year 1.06, 95% CI 1.05 to 1.07, *P *< 0.001). The proportion of legionellosis cases with positive direct fluorescent antibody (DFA) testing, or positive serological testing by paired serology [23], also declined over time (OR for DFA 0.78, 95% CI 0.60 to 0.85; OR for paired IFA 0.95, 95% CI 0.92–0.94, *P *for both comparisons < 0.001). Seropositivity reported as a result of a single high-titre positive IFA test was uncommon throughout the study period (Figure 4).

### Legionella Speciation

Data on definitive *Legionella *speciation were available for 351 cases with culture confirmation. Tentative species identification, based on direct and indirect immunological methods, was available for serum, tissue, and urine specimens from 533 additional cases. The distribution of species for culture confirmed isolates was significantly different for culture confirmed cases, as compared to those without culture confirmation (χ^2 ^squared 96.9 on 8 *d.f*., *P *< 0.001) (Table 2). Because of heterogeneity in the distribution of species depending on the presence or absence of culture confirmation, we restricted our subsequent primary analyses to culture-confirmed cases.

Among culture-confirmed cases, 10 distinct species of *Legionella *were represented, as well as 9 distinct serogroups of *Legionella pneumophila. L. pneumophila *serogroup 1 (LP1) was the most commonly isolated strain, with 66% of all isolates identified as LP1. LP6 and other strains of LP comprised an additional 22% of isolates with the remainder of cases associated with non-*pneumophila *species (Figure 4). Among 533 cases with tentative speciation based on immunologic methods, a smaller proportion (52%) was identified as LP1, and a greater proportion (36%) were identified as non-*pneumophilia *species.

When we evaluated predictors of the isolation of LP1 as opposed to other *Legionella *strains, in 351 culture-confirmed cases, Toronto and the contiguous Central East OHR isolates were most likely to have LP1 speciation (OR for Toronto 1.82, 95% C.I 1.14–2.90, *P *= 0.01; Central East OHR 4.06, 95% C.I 1.14–14.40, *P *= 0.03). An increased likelihood of LP1 speciation was seen between 1988 and 1997, compared to other decades (OR 1.73, 95% C.I 1.09 to 2.73, *P *= 0.02). No differences were seen with respect to gender or age and likelihood of LP1 species.

## Discussion

The centralized nature of testing for legionellosis in the Canadian province of Ontario allowed us to evaluate trends in this disease over a period of approximately 30 years. To our knowledge, this represents the longest time series of legionellosis cases in the biomedical literature, dating to shortly after the initial identification of *L. pneumophila *as a human pathogen [24]. Our findings exhibit similarities with previously published analyses of legionellosis epidemiology, but also exhibit important differences. We affirmed the strong associations between male gender and advanced age and legionellosis risk that have been described previously [11,12,25,26], and found strong evidence for summer-autumn seasonality of legionellosis in this region of Canada. Such seasonality has been observed in studies from other developed countries, and may suggest that seasonal environmental influences on environmental reservoirs (such as surface waters) influence human disease risk [12,25,27,28].

However, in this study we identify two features of legionellosis epidemiology in Ontario that appear to be at odds with observations in other jurisdictions: first, there is an apparent recent decrease in disease incidence; the second difference relates to the distribution of *Legionella *species, and (within the *L. pneumophila *species) bacterial serogroups associated with disease occurrence. Our identification of a statistically significant overall decrease in legionellosis incidence in the province since 1998 (notwithstanding an extremely large outbreak that occurred in the province in 2005 [17]), is at variance with reported increases in legionellosis reported recently in other North American jurisdictions, and in Australia and Europe [12,25,26,29].

An important question raised by investigators in these jurisdictions has been whether legionellosis incidence is truly increasing, or whether changes in test practices (particularly the use of urine antigen testing) have increased identification of this historically under-diagnosed infection. In a similar manner, differences in estimated legionellosis burden and trends in Ontario, relative to these other jurisdictions, could reflect true epidemiological differences, differences in regional testing practices (and disease reporting) [30], or differences in importation of travel-related legionellosis cases [31-33]. Other explanations for a true change in the burden of legionellosis in Ontario during the study period are also possible; for example, Canadian standards for construction and maintenance of hospitals and other public buildings were updated between 1999 and 2001 [34]; such changes may also have contributed to the amelioration of legionellosis risk observed during the last third of our observation period.

Indirect evidence suggests that differences in testing practice (particularly differences in the uptake of urinary antigen testing) are not primarily responsible for the difference in observed disease trends in Ontario, relative to other jurisdictions. As described above, there was a marked increase over time in the likelihood that Ontario legionellosis cases were identified by urine antigen testing, with or without other complementary testing methods. By contrast, the contribution of serological testing to diagnosis decreased markedly during the period under observation, and culture testing to diagnosis has changed in a non-linear fashion over time: we postulate that increases in the likelihood of culture-based diagnosis in our laboratory from the late 1980s to late 1990s may have reflected increasing technical skill over time, whereas declining probability of culture confirmation since 1998 may be attributable to changes in the use of urinary antigen testing mentioned above. In any case, the trends we describe here were robust when we restricted our analyses to cases with or without culture confirmation.

If the decline in legionellosis in Ontario reported here represents a true epidemiological shift, rather than an artifact of clinical practice patterns and laboratory testing practices, it may be worth noting that almost all major population centers in Ontario (Ottawa being the exception) lie < 30 miles from the Great Lakes. As *Legionella *species are abundant in surface waters, and the Great Lakes ecosystem is currently undergoing rapid ecological change [35], the possibility that downward trends in legionellosis relate to changes in local hydrology warrants further investigation. Although the proximate source of legionellosis is usually water distribution systems or cooling-tower related aerosols, the seasonality of legionellosis, described here and elsewhere [12,36], is also strongly suggestive of environmental influences on legionellosis risk. We have recently demonstrated that acute changes in local watershed hydrology in Toronto are linked to changes in legionellosis risk [37]. These associations warrant further investigation.

A second important difference between the epidemiological data reported here and those reported from other jurisdictions was the identification of *L. pneumophila *1 (LP1) in < 70% of cases with definitive speciation via culture methods (though exclusive use of culture-based methods may also favor identification of *L. pneumophila *species [38]); LP1 was identified in an even smaller proportion of cases with tentative speciation based on immunodiagnostic methods without culture confirmation. By contrast, the proportion of legionellosis cases attributable to LP1 has been reported to be > 90% in studies of legionellosis performed elsewhere in North America and Europe [39-41]. This may suggest that the parallel use of type-specific immunodiagnostic methods (such as serum IFA and tissue DFA, which are used in parallel with urine antigen testing and culture test methods at our laboratory whenever appropriate specimens are available) are important in the elucidation of the true diversity of pathogenic *Legionella *species, and that exclusive reliance on urine antigen testing methods may artificially inflate the proportion of cases due to LP1. Alternatively, this finding may again suggest that the epidemiology of legionellosis in this North American region is unique, and may be heavily influenced by the proximity of major population centers to the Great Lakes.

As might be expected given the geographic and demographic size and diversity of Ontario, we found evidence for important within-province heterogeneity in disease epidemiology as well. Disease incidence in the Eastern OHR (where Ottawa represents the major population aggregation) was approximately one tenth that seen in the Toronto OHR. Although some clinical laboratory urine antigen testing for legionellosis is performed in tertiary care centers in Ottawa and Kingston, the magnitude of this difference may, again, suggest that proximity to the Great Lakes is an important modulator of disease epidemiology.

Like any observational study, this study has important limitations. Principle among these is our use of a laboratory database for epidemiological purposes; this has the effect of inflating case estimates for regions with high concentrations of tertiary care centers (such as Toronto), as geographic coding reflects the locale of the healthcare institution that submitted patient specimens for testing, rather than patients' home addresses, and also means that desirable covariates (such as immune compromise, smoking status, comorbid illnesses, and case outcomes) are unavailable. Nonetheless we do not believe that such reporting effects would explain the magnitude in observed difference in disease burden between Toronto and the Ottawa region (itself a major site of tertiary care beds in the province). Furthermore, we believe that the wide geographic base, prolonged interval of data collection, detailed information on testing practices, and performance of all testing by a single laboratory distinguish this study from those of shorter duration conducted in Europe and the U.S. [11,26], and from previous Canadian sentinel surveillance efforts [13], and also provide new insights into the distinctive epidemiology of this disease in Canada's most populous province.

## Conclusion

In conclusion, we evaluated trends in legionellosis case occurrence and use of diagnostic modalities from the vantage of a major public health service laboratory over a period of three decades. While observed legionellosis epidemiology in this jurisdiction bore some similarities to that described in other jurisdictions, the apparent decline in legionellosis in the past decade in Ontario, and the wide variety of *Legionella *species identified in association with illness, suggest that the epidemiology of legionellosis in this North American region may be distinctive. Molecular epidemiologic studies in progress are likely to provide further insights into the phylogenetic characteristics of pathogenic *Legionella *species in Ontario.

## Competing interests

The authors declare that they have no competing interests.

## Authors' contributions

VN and DF contributed to study concept and design, analysis and interpretation of data. VN drafted the manuscript and all other authors were involved in critical manuscript revision. PT was involved in primary testing of specimens and test development, creation of the study database, and data analysis and interpretation. FJ, CG, and DEL contributed to laboratory testing and test development. All authors meet standard criteria for authorship.

## Pre-publication history

The pre-publication history for this paper can be accessed here:

http://www.biomedcentral.com/1471-2334/9/68/prepub
